# Longitudinal In-Bed Pressure Signals Decomposition and Gradients Analysis for Pressure Injury Monitoring

**DOI:** 10.3390/s21134356

**Published:** 2021-06-25

**Authors:** Nasim Hajari, Carlos Lastre-Dominguez, Chester Ho, Oscar Ibarra-Manzano, Irene Cheng

**Affiliations:** 1 Multimedia Research Centre, Department of Computing Science, University of Alberta, Edmonton, AB T6G 2E8, Canada; locheng@ualberta.ca; 2 Department of Electronics Engineering, Universidad de Guanajuato, Salamanca 36885, Mexico; cm.lastredominguez@ugto.mx (C.L.-D.); ibarrao@ugto.mx (O.I.-M.); 3 Department of Medicine, University of Alberta, Edmonton, AB T6G 2E8, Canada; chho@ualberta.ca

**Keywords:** pressure injury, in-bed pose estimation, signal filtering and analysis, pressure tracking

## Abstract

Pressure injury (PI) is a major problem for patients that are bound to a wheelchair or bed, such as seniors or people with spinal cord injuries. This condition can be life threatening in its later stages. It can be very costly to the healthcare system as well. Fortunately with proper monitoring and assessment, PI development can be prevented. The major factor that causes PI is prolonged interface pressure between the body and the support surface. A possible solution to reduce the chance of developing PI is changing the patient’s in-bed pose at appropriate times. Monitoring in-bed pressure can help healthcare providers to locate high-pressure areas, and remove or minimize pressure on those regions. The current clinical method of interface pressure monitoring is limited by periodic snapshot assessments, without longitudinal measurements and analysis. In this paper we propose a pressure signal analysis pipeline to automatically eliminate external artefacts from pressure data, estimate a person’s pose, and locate and track high-risk regions over time so that necessary attention can be provided.

## 1. Introduction

Pressure injury (PI) development is a chronic condition caused by the blockage of the blood flow due to prolonged interface pressure between skin and the supporting surface such as a bed or wheelchair. Seniors populations and patients with spinal cord injuries are the more high-risk groups to develop PI. As [1] mentioned, there have been over 2.5 million patients with PI conditions in the US in 2020. In total, 29,000 cases of death from PI have been reported in 2013, which is more than 100% increase from 1990 [2]. The prevalence of this condition is much higher in the Canadian healthcare settings (26%) compared to European health care settings (2%) [3]. Data from the Agency for Healthcare Research and Quality show that the rate of pressure injuries in the United States rose by 6% from 2014 to 2017 [4]. These are just some samples of the problem in today’s world. Although PI can be life threatening in its later stages, properly monitoring and assessing the longitudinal surface pressure can decrease the chance of developing PI significantly. The current clinical method of interface pressure monitoring is limited by periodic snapshot assessments, without longitudinal measurements and analysis. Despite all the efforts to effectively monitor in-bed body pressure and predict the chance of developing PI, it still remains a challenging healthcare and research problem to date.

A possible solution to prevent the development of pressure injury is the use of a pressure mattress. The idea is to reduce the blood blockage by distributing the pressure more evenly over the body or changing the patient’s pose regularly. A pressure *monitoring* mattress is another possible solution. As a previous study suggests [5], pressure monitoring mattresses can reduce the risk of developing PI significantly. These devices provide clinicians and patients with valuable body pressure information to locate the high pressure areas so that caregivers can change the body pose effectively. However, the focus of such systems are on spatial pressure data analysis and they lack the temporal analysis. This can lead to the same skin surface, such as the sacrum, to be under accumulated pressure for an extended amount of time. Having a computer-assisted system to perform longitudinal analysis, automatic body pose estimation, and body part location and tracking would be very beneficial in detecting the high-risk regions, and monitoring them over time, so that caregivers can adjust the patient’s posture effectively. The existence of the external objects on the pressure mattress, e.g., pillows and wedges, can distort the pressure signal and make the automatic analysis inaccurate.

A number of techniques for automatic human pose estimation from optical images and videos have been proposed in recent years with the help of artificial neural networks (ANN). Pose estimation networks such as OpenPose [6], Hourglass [7], ResNet [8], and HRNet [9] are regarded as the backbones for the state-of-the-art techniques; however, applying these networks to pressure map images, which are generally noisy, of low resolution, and without textural information or other visual cues, requires network modification and tremendous amount of labelled training data.

We propose a novel method to remove external objects from the pressure signals, automatically detect the in-bed body postures, detect and label high-risk body regions, and track them over time. As our experiments show, the pressure signals generated by the body and the external objects have different statistical characteristics. We use these characteristics to clean the pressure signals and eliminate the external objects from the body silhouette. To the best of our knowledge, this is the first work that removes external objects from pressure signals. Related works assume that the body silhouettes are fully or noticeably detectable in the pressure signal maps. We train various classifiers on the histogram of oriented gradient (HOG) features of gradient vector field (GVF) of the pressure signals to classify the in-bed poses into back, right lateral, and left lateral. Finally, we detect and track the high-risk regions for each pose over time. The high-risk regions are head, shoulders, sacrum, and feet. Our work has a number of major contributions:

1. We introduce a robust algorithm to separate the occlusion imposed by external objects from the body pressure signals.
2. Detect body part for high-risk regions including head, sacrum, shoulders, and feet, directly from the spatial pressure distribution. Unlike previous works, we do not need any model fitting or multi-modality pose estimation networks.
3. Track the high-risk regions and the corresponding pressure values over time. This is an essential step to formulate PI development as the major factors causing PI are (a) the surface pressure, (b) how long the pressure is exerted, and (c) the skin resistance to the building pressure.

The remainder of this paper is organized as follows: Section 2 discusses the related work of in-bed pose estimation and pressure injury prevention. The details of the proposed method are discussed in Section 3. The details of the dataset and experimental results are presented in Section 4. Finally, Section 5 concludes the paper.

## 2. Related Work

Pressure data analysis for in-bed pose estimation has been studied in the literature for patient monitoring, PI prevention, and sleep analysis. Researchers used edge and contour information, skeleton mapping, and pose estimation networks to detect in-bed postures, limb detection, and pose estimation.

Conventional in-bed pose estimation methods from pressure data are based on feature extraction and a learning-based classifier. Grimm et al. [10] proposed a system to automatically classify the pose into prone, supine, left, and right using a K-nearest neighbour (KNN) classifier. They also used an optimization method to fit a human body model related to that pose into the observed pressure data. They applied a heuristic to differentiate the pressure distribution in the generic model. Hsia et al. [11] estimated the in-bed posture based on the pressure distribution of the upper body. They used a bed with 16 force-sensing resistors (FSR), located on the top part of the bed. They extracted kurtosis and skewness from the pressure distribution and used a Bayesian classifier to detect posture. Youseffii ett al. [12] used principal component analysis (PCA), independent component analysis (ICA), and a KNN classifier to detect the in-bed posture from pressure data. Ostadabbas et al. [13] used Gaussian mixture model (GMM)-based clustering approaches for posture classification and limb identification. Some researchers used descriptors such as histogram of oriented gradients (HOG) and scale invariant feature transform (SIFT) with support vector machines (SVM) and other classifiers to classify the in-bed postures [14,15]. Skeletonization-based pose estimation is another technique used by [16,17]. Others used pictorial structures to estimate the body part locations based on the appearance and spatial information in the pressure image [18]. Baran et al. [19] created a public dataset, called pressure map, as the result of two separate experimental sessions. They collected the pressure data from 13 participants in various poses with and without external objects. Although the number of participants in this experiment is small, and the recording length for each posture is short, this is a reasonable and comprehensive dataset to generate an in-bed pose estimation model.

An artificial neural network (ANN), used to estimate in-bed postures from pressure data, was recently proposed in the literature [20,21,22,23]. Some of these techniques adapted a heavy pre-processing step to ensure the pressure data as close as possible to the optical data and then used a pre-trained pose estimation network to estimate in-bed posture from pressure data [21]. The performance highly depends on the quality of the pressure data. Noisy and low-resolution data can reduce the accuracy significantly. Other ANN approaches build networks from scratch or retraining the available networks with pressure data [20,23]. These approaches require a representative labelled training set, which can be difficult to obtain, especially if a second modality of data, such as optical videos [24] or motion capture (MoCap) data are needed [25,26]. Clever et al. [26] proposed a system with two convolutional neural networks (CNN) to estimate the 3D joint positions of a person in a configurable bed setting. To train the network, the authors collected MoCap information by connecting MoCap sensors to the body of the subjects. After training the network, the system can then estimate the 3D joint positions or kinematic model from a single pressure image. However, the variations in height and weight between the training and test subjects can create errors in estimating joint positions, especially for joints with smaller pressure values. Matar et al. [23] proposed a human body lying posture (HBLP) system. They extracted HOG, local binary patterns (LBP), and body weight distributions and fed them to a supervised ANN for classification.

## 3. Proposed Method

**Figure 1:**
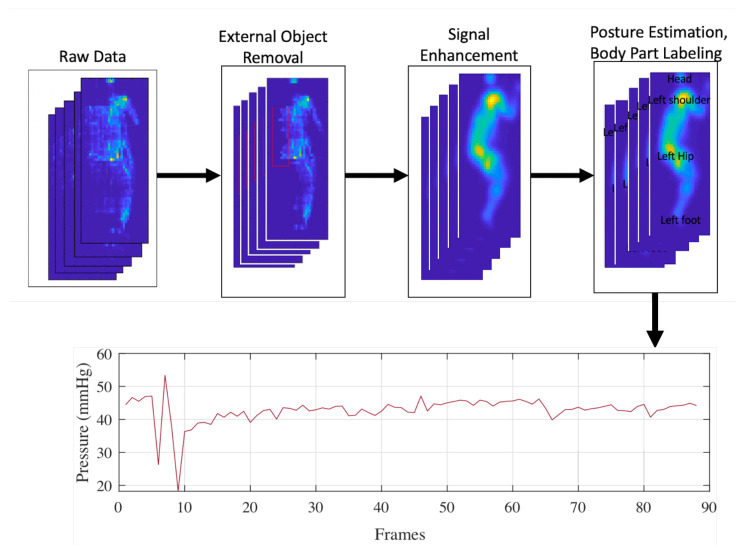
The overview of the proposed system pipeline.

In this paper, we propose a novel method to clean and enhance the pressure signal, automatically estimate the in-bed body posture, and finally detect the high-risk body parts and track them over time. We use the statistical characteristics of the pressure signals (signal trends) to remove the external objects and enhance the signals. HOG features are extracted from the GVF of the pressure signals and SVM and RF classifiers are used to classify the in-bed postures. The postures were classified into back, right lateral, and left lateral as they are the most relevant in-bed postures for the elderly and patients with spinal cord injuries. Finally, the high-risk body regions are detected and tracked over time. These regions are head, shoulders, sacrum, and feet. Figure 1 shows a high-level overview of the proposed system and the results of different steps.

### 3.1. Pre-Processing of the Pressure Data

The captured pressure data from the pressure mattresses are usually noisy and of low resolution. More specifically, the existence of the external objects, such as pillows or wedges, on the mattress can affect the accurate pressure recording. Pillows and wedges are used to provide extra support for the patients. Usually these external objects can change the pressure signal distribution, the body silhouette, and make the pose estimation and body part detection and tracking quite challenging. Further, eliminating the external objects from the pressure signals is quite challenging as their corresponding effects will be different depending on the patient’s body structure, location, and characteristics of the external objects. To the best of our knowledge this is the first work that removes the external object from the pressure signals based on trend analysis of human body pressure distribution and external object pressure distribution. More details on the external object removal and signal enhancement are presented in Section 3.1.1 and Section 3.1.2.

#### 3.1.1. External Object Removal

**Figure 2:**
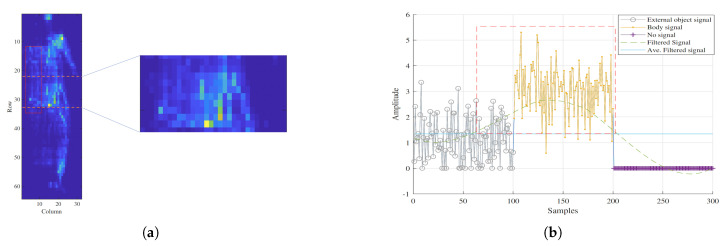
(**a**) Pressure image for a subject on the bed with a wedge. The wedge is inside the red rectangle. The details of the pressure signal for the area inside the dashed lines is shown in (**b**). (**b**) Pressure signal distribution for the wedge (gray signal), body (orange signal), and nothing on the pressure mattress (purple signal). The green dashed curve shows the Savitsky–Golay smoothing filter and its average value is shown in blue line.

The intuition behind our external object removal approach is that body pressure distribution shows very distinctive and different characteristics comparing to the external object pressure distribution. Figure 2 shows the pressure signal distribution (the 2D pressure signal is unfolded into 1D) for external object, body, and empty space. The region of interest is shown inside the dashed red rectangle (Figure 2b).

As Figure 2b shows, each part of the signal has a different trend. Here, the trend caused by the body is the most representative one. To capture the trend of a signal, the most common approaches are moving average (MA) and Savitsky–Golay filters [27]. The latter keeps the local characteristics, such as local minima and maxima, of the signal more effectively and is therefore more appropriate to capture the trend of a mixed signal. To keep the main component of the signal (pressure values related to patient’s body) and eliminate the unwanted regions, we applied the Savitsky–Golay filter, proposed by [28].

The Savitsky–Golay filter is defined as a weighted moving average where weights are polynomial functions of specific degree (*k*) over a window of size *N*. Given a window determined by 2M+1 samples centred at n=0, we represent the coefficients of a polynomial form given by Equation (1).

(1) $$p\left(n\right)=\sum_{i=0}^{N}a_{k}n^{i}$$

which minimizes the approximated mean-square error, ε, for 2M+1 samples centred at n=0:

(2) $$\varepsilon =\sum_{i=0}^{M}{\left(p\left(n\right)-x\left[n\right]\right)}^{2}$$

For each point *n*, the smoothed output value provided by sampling the fitted polynomial is identical to a linear combination of the local set of input samples. In other word the 2M+1 input within the approximation interval are combined using a set of weighted coefficients that can be calculated once for a $k^{th}$ order polynomial with length interval 2M+1. The output samples can be calculated by a discrete convolution form given by Equation (3).

(3) $$S^{\prime }\left[n\right]=\sum_{m=-M}^{M}\left(h(n)-x[n-m]\right)$$

where *h* represents the impulse response needed to compute the estimation of the input signal ($S^{\prime }\left[n\right]$). In order to find an impulse response equivalent to the least-squares polynomial smoothing for the mentioned interval, we need to find the polynomial optimal coefficients by differentiating Equation (2) with respect to each of the k+1 unknown coefficients as presented in Equation (4).

(4) $$\begin{array}{cc} \frac{\partial \varepsilon }{\partial a_{j}} & =\sum_{n=-M}^{M}2n^{j}(\sum_{l=-0}^{k}a_{l}n^{l}-x\left[n\right])=0\\ \; & =\sum_{l=0}^{k}\sum_{l=-M}^{M}n^{j+l}a_{l} \end{array}$$

The above equation can be represented in matrix form as:

(5) $$A^{T}\mathrm{Aa}=A^{T}x$$

where A is defined by:

(6) $$A=\left[\begin{array}{ccccc} 1 & n_{0} & n_{0}^{2} & \cdots & n_{0}^{k}\\ 1 & n_{1} & n_{1}^{2} & \cdots & n_{1}^{k}\\ \vdots & \vdots & \vdots & \ddots & \vdots \\ 1 & N & N^{2} & \cdots & N^{k} \end{array}\right]$$

where x=${\left[x\left[-M\right],\cdots x\left[M\right]\right]}^{T}$. Considering that $H={\left(A^{T}A\right)}^{-1}A^{T}$, the coefficients of a formulated as:

(7) $$a={\left(A^{T}A\right)}^{-1}A^{T}x\overset{\Delta }{=}\mathrm{Hx}$$

The matrix $H=a={\left(A^{T}A\right)}^{-1}A^{T}$ with the dimension (2M+1)×(k+1) is denoted by the impulse responses, where H depends only on *k* and *M*.

In this work a polynomial function of a low degree (k=2 or k=3) is adequate to capture the trend of the signal. Further, based on our experiments, to better capture the trend, the window size should be $M=\frac{r}{2}\pm \epsilon$—where *r* is the the number of the pressure sensors in the horizontal direction and ϵ depicts a small adjustment to the window size depending on the type and number of external objects. This approach smooths the signal and highlights its trend.

**Figure 3:**
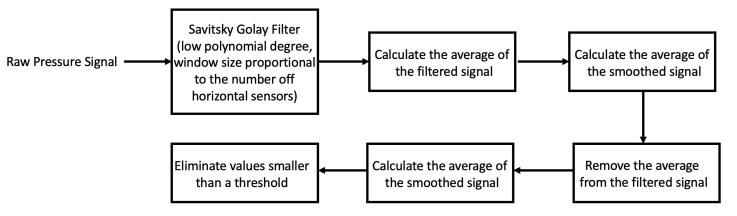
The proposed pipeline to remove external object from pressure signal.

The next step is removing the smoothed average from the smoothed signal and eliminating any values smaller than a threshold. The remaining signal corresponds to the body part pressure distribution. Equation (8) summarizes this procedure.

(8) $$S^{\prime }=\left|f\left(S\right)-avg\left(f\left(S\right)\right)\right|,\;b=S⊙\left(S^{\prime }>th\right)$$

where *S* is the original raw pressure signal, *f* is the smoothing filter determined by (3), th is the threshold, and *b* is the cleaned body pressure signal. ⊙ is the element-wise multiplication of the two arrays. The threshold value is found through a grid search approach. Figure 3 summarizes our proposed algorithm for external object removal.

**Figure 4:**
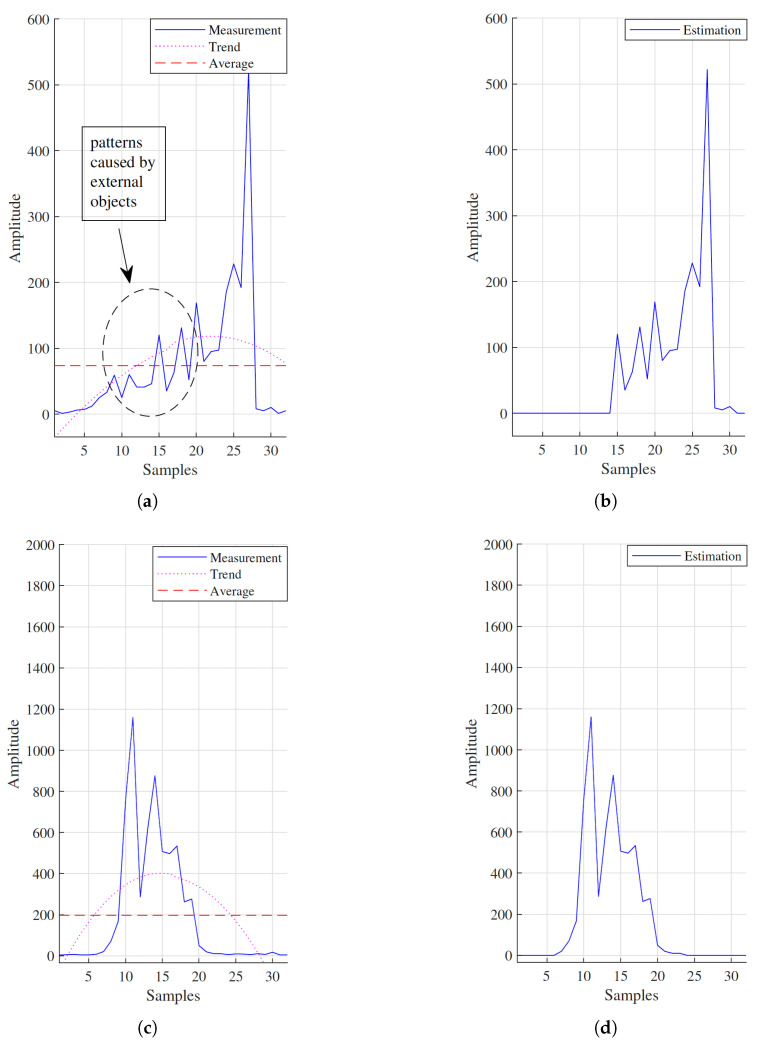
(**a**) Pressure signal for one column of the sensor, where an external object is presented. Solid plot represents the raw input signal. The dot curve shows the trend of the signal (the filtered signal) and the dashed line is its average. (**b**) Estimation of the corresponding body signal. (**c**) Pressure signal for one column of the sensor with no external object. Solid plot represents the raw input signal. The dot curve shows the trend and the dashed line is the average of the filtered signal. (**d**) Estimation of the corresponding body signal. Both original and estimated body signal are identical.

Figure 4a,b shows how our proposed approach removes the external object from the pressure signal. It also shows that when there are no external objects on the pressure mattress, this approach does not have any effect on the original signal (Figure 4c,d).

More results and in depth analysis are presented in Section 4. Note that the proposed method is based on the entire signal trend and it would be challenging to separate different components of the signal, if the existence of the external objects alters the main signal trend significantly. This can happen if there are numerous external objects on the mattress.

#### 3.1.2. Signal Enhancement

**Figure 5:**
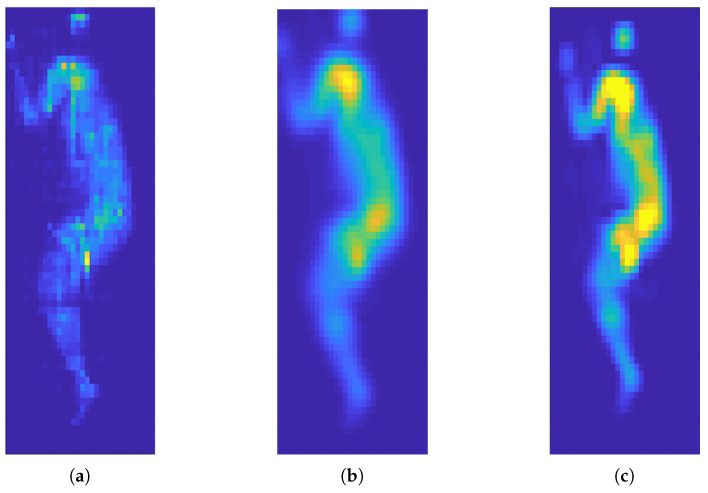
Smoothing results on frame 20 for a subject in right lateral position with few various filters. (**a**) Raw data, (**b**) Gaussian filter with σ= 1.4, and (**c**) Savitsky–Golay of degree 2 and window size 6 are shown.

To remove the system noise and smooth and enhance the pressure signal, we have tried different smoothing filters, including uniform, circular, pyramidal, conical, binomial, Gaussian, and Savitsky–Golay, as described in our previous work [29]. The qualitative analysis shows that Gaussian and Savitsky–Golay filters provide a better results in eliminating the noise and maintaining the local characteristics of the signal. More specifically, the Gaussian filter smooths out the whole signal, which creates a better visualization and silhouette of the body; whereas the Savitsky–Golay filter keeps the local maxima and minima and therefore provides more reliable signal analysis. Figure 5 shows the raw data of a subject in a right lateral position and the result of a Gaussian and Savitsky–Golay filter.

### 3.2. Automatic Posture Detection

**Figure 6:**
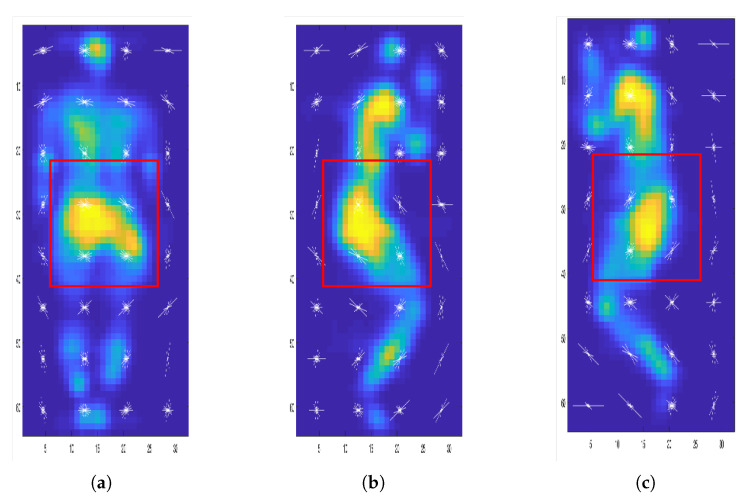
Extracted histogram of oriented gradient (HOG) for one subject in three different postures: (**a**) supine, (**b**) left lateral, and (**c**) right lateral. Classification is based on the whole extracted features aas well as the extracted features inside the red box.

People have proposed different approaches for in-bed posture detection or full-body pose estimation. These methods can be categorized into two main groups: (1) posture detection based on feature descriptors, such as [11,12,14,15]; (2) pose estimation based on skeleton methods [16,17,20,21,23]. In this work we classify the posture into supine and left and right lateral. We adapt the first paradigm and extract the HOG features [30] from pressure images. We classified the postures based on the HOG features of the whole body as well as the HOG features of the sacrum area only. Our findings show that although the sacrum is always in contact with the pressure mattress (for the three mentioned postures), posture estimation based on sacrum features only is not as accurate as the whole body features. Figure 6 shows the extracted HOG for three different postures.

### 3.3. Automatic Body Part Detection

As we explained in [29], the high-risk regions that are more prone to developing pressure injuries are the head, shoulders, sacrum, and feet. Usually these areas have a higher interface pressure compared to the neighbourhood regions as shown in Figure 6. Therefore, we propose a method to detect these parts using local maxima. We first compute the gradient field of the pressure image and then use zero crossing to find local maxima in the 2D space.

**Figure 7:**
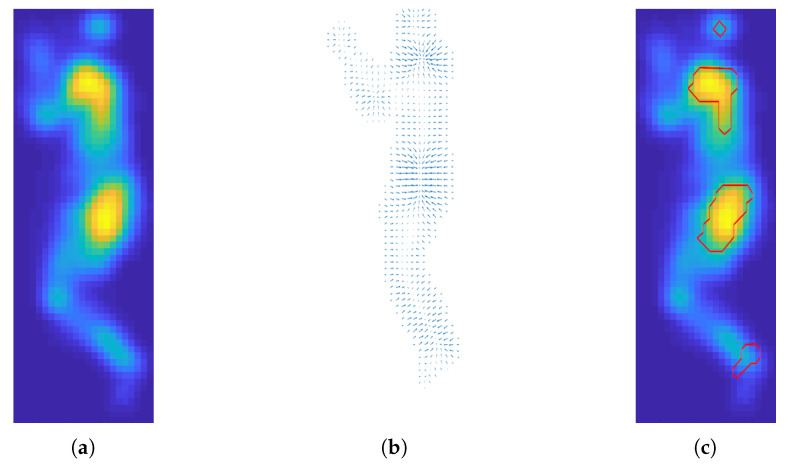
(**a**) Cleaned and enhanced pressure image after using Savitsky–Golay and Gaussian filter. (**b**) Gradient vector obtained from the pressure image. (**c**) The detected high-risk body parts are shown in red.

The gradient is given in Equation (9). Note that $\hat{i}$ and $\hat{j}$ are the unit vectors in the direction of the *x* and *y* coordinates, respectively. The gradient field is shown in Figure 7b.

(9) $$\nabla \left(f\right)=\frac{\partial f}{\partial x}\hat{i}+\frac{\partial f}{\partial y}\hat{j}$$

We use relative location information and morphological operations to remove small detected regions such as hands and elbows which are not at higher risks of developing PI.

**Figure 8:**
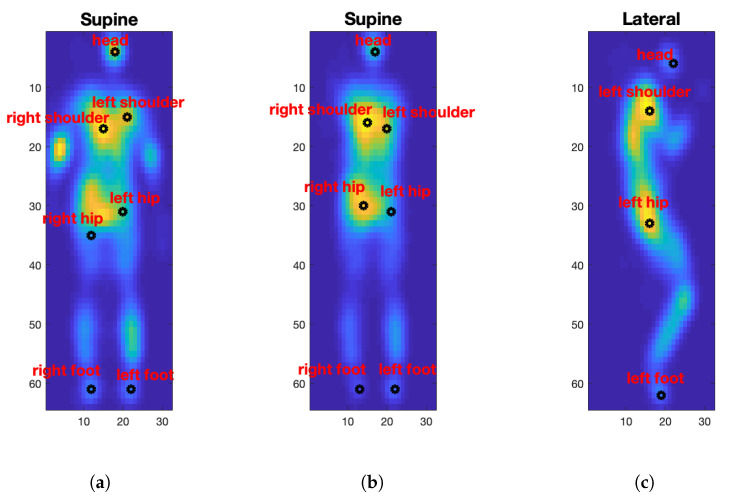

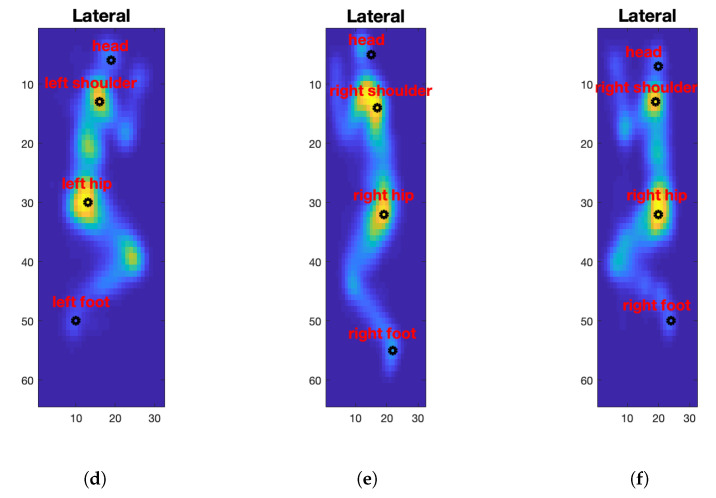
Labelled body parts for a (**a**,**b**) supine posture, (**c**,**d**) left lateral, and (**e**,**f**) right lateral.

We use a simple spatial relation approach to label the detected regions automatically and accurately. The intuition is that for monitoring patients who cannot move effectively on the mattress because of physical disabilities, we can assume that body position and orientation will remain unchanged. Figure 8 shows the detected and labelled body regions for different postures. The reported pressure value for each detected body part is the average pressure value of the corresponding extracted region.

## 4. Experimental Setup and Results

We used the PmatData [19], which is a publicly available pressure imaging dataset for in-bed pose estimation. It contains pressure data of two separate experiments. We tested our algorithm on the data collected from the first experiment, as the setup is closer to that of the healthcare settings. Data were collected using Vista Medical FSA SoftFlex 2048. There are 32×64 pressure sensors in the mattress and the range of capture is [0,1000] mmhg. The sampling is 1Hz. In total there are 13 subjects with an age range of [19,34], a height range of [169,186] cm, and a weight range of [63,100] kg. There are three main postures: supine, left lateral, and right lateral. However, the variations in bed inclination, adding external wedges, and arms and legs position results in 17 different poses. The pressure data for each subject and pose were captured for around 2 min. Although there are a limited number of subjects in this experiment, the variations in subjects characteristics (13 subjects in total) and the duration of the experiment for each subject (approximately 2 min with 1 Hz sampling rate) and each pose (17 poses) generates a reasonably large dataset to work with and validate the algorithm performance.

**Figure 9:**
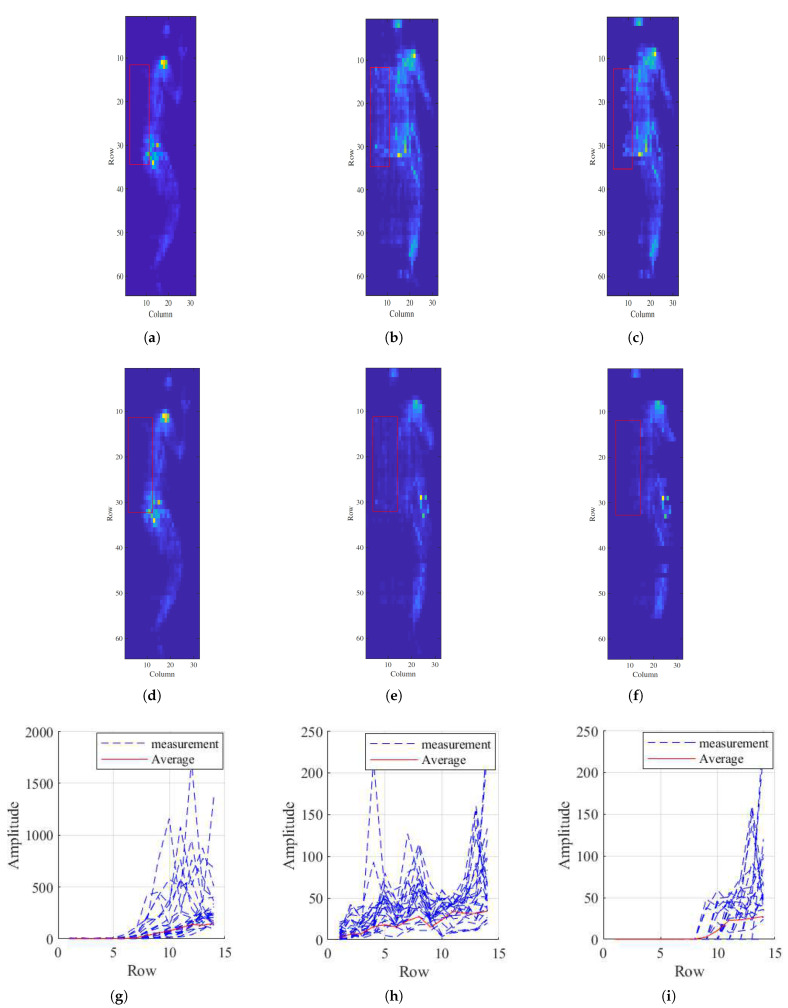
(**a**,**d**) A subject on pressure mattress without an external object. (**b**) The same subject and posture with one external object. (**e**) The same subject and posture with two external objects. (**c**,**f**) The cleaned pressure image after removing the external objects. (**g**–**i**) The close up of signals inside the red box of (**d**–**f**), respectively.

Some results of the proposed external object removal approach for two different scenarios (one and two external objects) are shown in Figure 9c,f, respectively. Figure 9g–i show the close up of signals inside the red box of Figure 9d, no external object, and (f), cleaned signal after removing external object, respectively. A comparison between the average curve of two figures shows that our method is very effective in removing the external objects. We also applied the quantitative analysis and the result is shown in the Table 1 where the average error is given by the difference between the pressure signal with no external object (considered the ground truth) and cleaned pressure signal after removing the external object.

We ran our experiment on a PC with Intel Core i5 processor and 8 GB of memory. We tried different classifiers, including support vector machine (SVM), K-nearest neighbour (KNN, k=10), and random forest (RF), to create a model to automatically detect the in-bed postures. Table 1 shows the accuracy per class for each evaluation metric, when trained on the whole body HOG features and HOG features of the sacrum area only. To reduce the chance of over-fitting, we used 10-fold cross validation in the training phase. We randomly selected 20% of the subjects in the experiments as the test subjects and train the models on the remaining 80%. As Table 2 shows, the classifiers trained on the HOG features of the whole body are more accurate than the ones trained on the HOG features of the sacrum area only. Further, the comparison results with the state-of-the-art techniques demonstrate the effectiveness of our proposed method.

**Figure 10:**
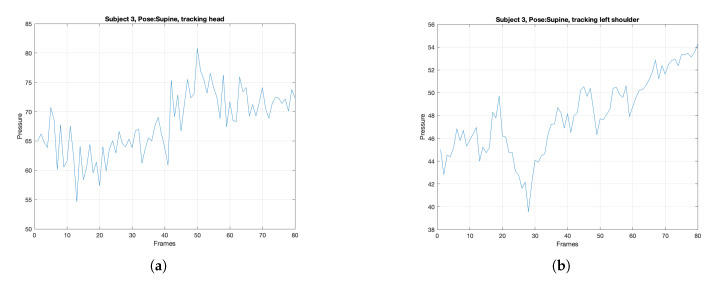

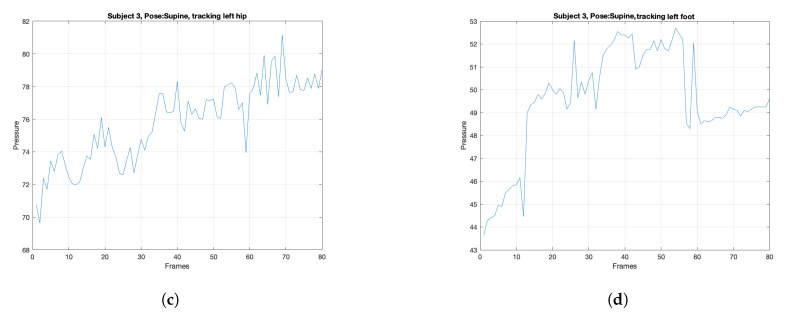
Tracking pressure values over time for a subject in supine position for (**a**) head, (**b**) left shoulder, (**c**) left hip, and (**d**) left foot.

We detected body parts for each frame and tracked the corresponding pressure value over time. Figure 10 shows the tracking plots for subject 3 in the supine position. Note that the reported pressure value for each body part is the average pressure value in each extracted region.

## 5. Conclusions

Monitoring in-bed pressure for patients with spinal cord injuries and elderly populations can reduce the chance of developing pressure injuries (PI). Developing PI depends not only on the interface pressure, but also on the time that the body parts are under pressure. Some body parts, including head, shoulders, sacrum, and feet, are more prone to PI development. One of the challenges in analysing pressure signals captured from the pressure mattresses is the existence of external objects such as pillows and wedges. We proposed a novel method to separate the pressure signal caused by external objects from the body’s pressure signal and automatically detect and track the high-risk regions from the processed cleaned and enhanced pressure data. Our experimental results show that this signal cleaning and enhancement is a crucial first step to accurately estimate the in-bed body pose and detect and track body parts over time. However, the proposed external object removal method requires the distinguishable trend of the main signal and existence of numerous external objects on the mattress will affect the efficiency of the proposed approach. Further, the experimental results show that the proposed pipeline is more accurate in body pose estimation compared to the state-of-the-art techniques. Tracking body parts over time is a crucial first step in analysing and predicting the chance of PI development. In the future we will consider other factors that are important in developing PI, such as skin resistance and blood oxygen level. We will develop a computational model to automatically capture the effect of all of these factors and incorporate it with our body part detection and tracking algorithm to better estimate the chance of PI development for each individual. 

## Figures and Tables

**Table 1:** Peak signal-to-noise ratio (PSNR) and root mean square error (RMSE) for different subjects after removing the external objects.

	S1	S2	S3	S4	S5	S6	S7	S8	S9	S10	S11	S12	S13
PSNR	16.13	15.34	14.57	14.74	19.58	17.34	16.04	19.26	16.88	17.93	15.08	18.65	14.23
RMSE	0.102	0.112	0.129	0.124	0.080	0.087	0.130	0.083	0.092	0.080	0.115	0.085	0.128

**Table 2:** Accuracy results of the posture estimation models on PmatData [19] for the HOG features of the whole body (WHOG) and sacrum area only (SHOG).

**WHOG**				
	**SVM**	**KNN**	**RF**	**SSRM [31]**
**Supine**	99.98%	99.99%	99.93%	99.90
**Right lateral**	99.99%	100%	99.98%	98.48
**Left lateral**	99.99%	99.99%	99.95%	99.57
**SHOG**				
	**SVM**	**KNN**	**RF**	**SSRM [31]**
**Supine**	99.95%	98.31%	99.91%	99.90
**Right lateral**	100%	98.21%	99.95%	98.48
**Left lateral**	99.95%	99.88%	99.95%	99.57

## Data Availability

Not applicable.

## References

[1] Mansfield S., Obraczka K., Roy S. (2020). Pressure Injury Prevention: A Survey. IEEE Rev. Biomed. Eng..

[2] Abubakar I., Tillmann T., Banerjee A. (2015). Global, regional, and national age-sex specific all-cause and cause-specific mortality for 240 causes of death, 1990-2013: A systematic analysis for the Global Burden of Disease Study 2013. Lancet.

[3] McInnes E., Jammali-Blasi A., Bell-Syer S.E., Dumville J.C., Middleton V., Cullum N. (2015). Support surfaces for pressure ulcer prevention. Cochrane Database Syst. Rev..

[4] Hospital Acquired Conditions Are Declining So Why Are Pressure Injuries on the Rise?. https://www.advisory.com/daily-briefing/2019/11/14/pressure-injury.

[5] Walia G.S., Wong A.L., Lo A.Y., Mackert G.A., Carl H.M., Pedreira R.A., Bello R., Aquino C.S., Padula W.V., Sacks J.M. (2016). Efficacy of monitoring devices in support of prevention of pressure injuries: Systematic review and meta-analysis. Adv. Ski. Wound Care.

[6] Cao Z., Martinez G.H., Simon T., Wei S.E., Sheikh Y.A. (2019). OpenPose: Realtime Multi-Person 2D Pose Estimation using Part Affinity Fields. IEEE Trans. Pattern Anal. Mach. Intell..

[7] Newell A., Yang K., Deng J. (2016). Stacked hourglass networks for human pose estimation. European Conference on Computer Vision.

[8] Szegedy C., Ioffe S., Vanhoucke V., Alemi A.A. Inception-v4, inception-resnet and the impact of residual connections on learning. Proceedings of the Thirty-First AAAI Conference on Artificial Intelligence.

[9] Sun K., Xiao B., Liu D., Wang J. Deep high-resolution representation learning for human pose estimation. Proceedings of the IEEE Conference on Computer Vision and Pattern Recognition.

[10] Grimm R., Sukkau J., Hornegger J., Greiner G. (2011). Automatic patient pose estimation using pressure sensing mattresses. Bildverarbeitung Für die Medizin 2011.

[11] Hsia C.C., Hung Y.W., Chiu Y.H., Kang C.H. Bayesian classification for bed posture detection based on kurtosis and skewness estimation. Proceedings of the HealthCom 2008-10th International Conference on e-health Networking, Applications and Services.

[12] Yousefi R., Ostadabbas S., Faezipour M., Farshbaf M., Nourani M., Tamil L., Pompeo M. Bed posture classification for pressure ulcer prevention. Proceedings of the 2011 Annual International Conference of the IEEE Engineering in Medicine and Biology Society.

[13] Ostadabbas S., Pouyan M.B., Nourani M., Kehtarnavaz N. In-bed posture classification and limb identification. Proceedings of the 2014 IEEE Biomedical Circuits and Systems Conference (BioCAS) Proceedings.

[14] Beltrán-Herrera A., Vázquez-Santacruz E., Gamboa-Zuñiga M. (2014). Real-Time Classification of Lying Bodies by HOG Descriptors. Lecture Notes in Computer Science (Including Subseries Lecture Notes in Artificial Intelligence and Lecture Notes in Bioinformatics).

[15] Cruz-Santos W., Beltrán-Herrera A., Vázquez-Santacruz E., Gamboa-Zúñiga M. Posture classification of lying down human bodies based on pressure sensors array. Proceedings of the 2014 International Joint Conference on Neural Networks (IJCNN).

[16] Farshbaf M., Yousefi R., Pouyan M.B., Ostadabbas S., Nourani M., Pompeo M. Detecting high-risk regions for pressure ulcer risk assessment. Proceedings of the 2013 IEEE International Conference on Bioinformatics and Biomedicine.

[17] Pouyan M.B., Birjandtalab J., Nourani M., Pompeo M.M. (2016). Automatic limb identification and sleeping parameters assessment for pressure ulcer prevention. Comput. Biol. Med..

[18] Liu J.J., Huang M.C., Xu W., Sarrafzadeh M. Bodypart localization for pressure ulcer Prevention. Proceedings of the 2014 36th Annual International Conference of the IEEE Engineering in Medicine and Biology Society.

[19] Pouyan M.B., Birjandtalab J., Heydarzadeh M., Nourani M., Ostadabbas S. A pressure map dataset for posture and subject analytics. Proceedings of the 2017 IEEE EMBS International Conference on Biomedical & Health Informatics (BHI).

[20] Casas L., Navab N., Demirci S. (2019). Patient 3D body pose estimation from pressure imaging. Int. J. Comput. Assist. Radiol. Surg..

[21] Davoodnia V., Ghorbani S., Etemad A. (2019). In-bed Pressure-based Pose Estimation using Image Space Representation Learning. arXiv.

[22] Davoodnia V., Etemad A. Identity and Posture Recognition in Smart Beds with Deep Multitask Learning. Proceedings of the 2019 IEEE International Conference on Systems, Man and Cybernetics (SMC).

[23] Matar G., Lina J.M., Kaddoum G. (2019). Artificial neural network for in-bed posture classification using bed-sheet pressure sensors. IEEE J. Biomed. Health Inform..

[24] Huang W., Wai A.A.P., Foo S.F., Biswas J., Hsia C.C., Liou K. Multimodal sleeping posture classification. Proceedings of the 2010 20th International Conference on Pattern Recognition.

[25] Harada T., Sato T., Mori T. Pressure distribution image based human motion tracking system using skeleton and surface integration model. Proceedings of the 2001 ICRA, IEEE International Conference on Robotics and Automation (Cat. No. 01CH37164).

[26] Clever H.M., Kapusta A., Park D., Erickson Z., Chitalia Y., Kemp C.C. 3D Human Pose Estimation on a Configurable Bed from a Pressure Image. Proceedings of the 2018 IEEE/RSJ International Conference on Intelligent Robots and Systems (IROS).

[27] Azami H., Mohammadi K., Bozorgtabar B. (2012). An Improved Signal Segmentation Using Moving Average and Savitzky-Golay Filter. J. Signal Inf. Process..

[28] Savitzky A., Golay M.J.E. (1964). Smoothing and differentiation of data by simplified least squares procedures. Anal. Chem..

[29] Dominguez C.L., Hajari N., Ho C., Manzano O.I., Cheng I. Human Body Parts Tracking from Pressure Data: Toward Effective Pressure Injury Assessment. Proceedings of the 2021 International Conference on Human-Computer Interaction (HCI).

[30] Dalal N., Triggs B. Histograms of oriented gradients for human detection. Proceedings of the 2005 IEEE Computer Society Conference on Computer Vision and Pattern Recognition (CVPR’05).

[31] Zhao A., Dong J., Zhou H. (2020). Self-supervised learning from multi-sensor data for sleep recognition. IEEE Access.

